# Reporting FDR analogous confidence intervals for the log fold change of differentially expressed genes

**DOI:** 10.1186/1471-2105-12-288

**Published:** 2011-07-15

**Authors:** Klaus Jung, Tim Friede, Tim Beißbarth

**Affiliations:** 1 Department of Medical Statistics, University Medical Center Göttingen, D-37099 Göttingen, Germany

## Abstract

**Background:**

Gene expression experiments are common in molecular biology, for example in order to identify genes which play a certain role in a specified biological framework. For that purpose expression levels of several thousand genes are measured simultaneously using DNA microarrays. Comparing two distinct groups of tissue samples to detect those genes which are differentially expressed one statistical test per gene is performed, and resulting *p*-values are adjusted to control the false discovery rate. In addition, the expression change of each gene is quantified by some effect measure, typically the log fold change. In certain cases, however, a gene with a significant *p*-value can have a rather small fold change while in other cases a non-significant gene can have a rather large fold change. The biological relevance of the change of gene expression can be more intuitively judged by a fold change then merely by a *p*-value. Therefore, confidence intervals for the log fold change which accompany the adjusted *p*-values are desirable.

**Results:**

In a new approach, we employ an existing algorithm for adjusting confidence intervals in the case of high-dimensional data and apply it to a widely used linear model for microarray data. Furthermore, we adopt a concept of different relevance categories for effects in clinical trials to assess biological relevance of genes in microarray experiments. In a brief simulation study the properties of the adjusting algorithm are maintained when being combined with the linear model for microarray data. In two cancer data sets the adjusted confidence intervals can indicate significance of large fold changes and distinguish them from other large but non-significant fold changes. Adjusting of confidence intervals also corrects the assessment of biological relevance.

**Conclusions:**

Our new combination approach and the categorization of fold changes facilitates the selection of genes in microarray experiments and helps to interpret their biological relevance.

## Background

When simultaneously testing a large number of hypotheses, a high number of false positive test results is expected. This applies particularly in the case of high-dimensional data, where the number *m *of features is much larger than the available sample size *n*. Therefore, raw *p*-values are adjusted in order to control a false positive rate, for example the *false discovery rate *(*FDR*). The *FDR *was introduced by Benjamini and Hochberg [1] as the expected proportion of false positives among all positive test decisions. A prime example of high-dimensional data are gene expression levels from DNA microarray experiments. A frequent microarray study design is the comparison of gene expression levels among two distinct groups of tissue samples, for example from wild types and mutated subjects [2], resulting in several thousand *p*-values, one per gene. These raw *p*-values are then increased by an adjusting algorithm to reduce the number of false positive detections. Additionally, expression changes between the two groups are quantified by some difference statistic such as the log fold change. The reason for a difference statistic instead of a ratio statistic is that gene expression data are usually log-transformed by one of several data preprocessing steps. Published microarray experiments, where the log fold change is accompanied by a confidence interval, are rare, and the interval limits are usually not adjusted for multiplicity. These unadjusted confidence intervals are not comparable to *FDR*-adjusted *p*-values. One reason for that might be that *FDR*-adjustment procedures are based on ordered *p*-values (so called *step-up *or *step-down *procedures), while confidence intervals cannot be ordered according their level of significance. Therefore, an algorithm presented by Benjamini and Yekutieli [3] for adjusting confidence limits analogous to the *p*-values is also based on the order of their corresponding *p*-values. A similar algorithm was introduced by Jung et al. [4] who studied adjusted confidence intervals for the fold change of protein expression levels. Although the latter algorithm produces adjusted confidence intervals which match their related adjusted *p*-values in the sense that they lead to the same test decision, it has the drawback of gene-specific confidence levels. In contrast, the algorithm of Benjamini and Yekutieli [3] uses the same adjusted confidence level for all genes.

In order to evaluate the performance of their algorithm for adjusting confidence intervals, Benjamini and Yekutieli [3] introduced the *false coverage-statement rate *(*FCR*), which, in the context of DNA microarray experiments, is defined as the expected proportion of true log fold changes not covered by their confidence interval among all genes that have been detected as differentially expressed. They use this rate instead of the *conditional coverage probability *(*CCP*), i.e. the portion of true log fold changes covered by their confidence interval among all genes detected as differentially expressed. Both, *FCR *and *CCP*, are defined to be zero in the case that the number of genes detected as differentially expressed is zero, too. It can be shown that the *CCP *is dependent of the size of the fold changes, while the *FCR *is independent. Thus, studying the non-coverage of confidence intervals is more reasonable than studying their coverage.

Before building confidence intervals, genes have to be selected by statistical tests. A popular method framework for detecting differentially expressed genes among two distinct groups of samples is given by the linear models proposed by Smyth [5]. These models pick up the ideas of Lönnstedt and Speed [6] who recommended to use a moderated *t*-statistic for testing the differential expression of each gene. They argue that a very small variance is expected for some genes, when testing thousands of genes simultaneously, though the difference of group means is inconsiderable for these particular genes. As a consequence, the classical *t*-statistic will become unreasonably large for these genes. Therefore, Smyth [5] employed an empirical Bayes approach where a prior distribution for the variance of genes is assumed and the observed standard errors of the estimated model coefficients are shrinked towards these prior values. In the case of two groups, the coefficient of the related linear model can be taken as an estimate for the log fold change. As a new approach, we use these estimates as well as their shrunken standard errors to construct confidence intervals for the fold change, and employ the algorithm of Benjamini and Yekutieli [3] to adjust these intervals to control the *FCR*. With this, we account for a problem mentioned by Efron [7] and Ghosh [8], that is the confidence intervals proposed by Benjamini and Yekutieli [3] tend to be too wide. Combining an adjusting algorithm with a multiple testing procedure was also proposed in the paper of Jung et al. [4]. This former proposal had, however, the above mentioned drawback of non-uniform confidence levels and only uses simple confidence intervals based on the assumption of normal distribution. Thus, we present now an extended methodology.

Building confidence intervals for the log fold change adjusted by this new combination method is a helpful step for assessing genes which might be biologically relevant. In order to further categorize genes according their potential biological relevance, we adopt a concept developed to assess the clinical relevance of observed effects in clinical trials [9]. According to this concept, genes are classified individually into one of four relevance categories, based on the location of their confidence intervals relative to the zero log fold change and a relevance threshold.

The outline of this article is as follows. In the methods section we describe the linear model of Smyth [5] for the case of a two group comparison. Next, the construction of unadjusted and adjusted confidence intervals is detailed, followed by a short description of their implementation in the free software R [10]. Afterwards, the concept of relevance categories is explained. In the results section, we present the results of a brief simulation study with which we evaluate the behavior of the *CCP *and the *FCR *in a two group comparison. The benefit of incorporating adjusted confidence intervals for the log fold change and of categorizing genes by their potential biological relevance is further illustrated by two examples of microarray data, featuring gene expression levels observed in lung and rectal carcinomas, respectively. Finally we close with a discussion and some conclusions.

## Methods

### Selection of differentially expressed genes

The most frequent design in microarray experiments is the comparison of expression levels in samples from two distinct groups. Following the models of Smyth [5], an estimate for the log fold change of gene *j *(*j *= 1,..., *m*) is given by the estimate of the model parameter ***β***_*j*_. In order to test the hypothesis that the log fold change for gene *j *is equal 0, i.e. *H*_0*j *_: *β_j _*= 0, Smyth [5] uses a moderated *t*-statistic

where  are the shrunken standard errors as mentioned in the introduction. The moderated *t*-statistic can be shown to follow a *t*-distribution with augmented degrees of freedom f*. Estimation of *β_j _*and the determination of f* is explicitly detailed in Smyth [5].

Consider the object fit to be the output of the model fit for a two-group comparison using the limma-package for the free software R. In the R-environment, the estimated coefficients, , can be obtained by

*>*beta = fit$coefficients[,2]

The vector of standard errors  can be created by the following line

*>*se = sqrt(fit$s2.post) * sqrt(fit$cov. coefficients [2,2])

At last, the degrees of freedom f* are obtained by

*>*dof = fit$df.prior + fit$df.residual[1]

With these data vectors, one can easily implement the unadjusted and adjusted confidence intervals detailed in the subsection below. An example R-code is also provided in additional file 1

Using this test statistic, a raw *p*-value *p_j _*can be derived for each gene *j*. In the context of microarray analyses, these raw *p*-values are usually adjusted to control the *FDR *at a pre-specified level (e.g. 5%). The most commonly used adjusting method is that of Benjamini and Hochberg [1], in the following denoted as BH-method, which allows for certain dependencies among the *m *hypotheses. This step-wise procedure orders first the *m *raw *p*-values by increasing size, i.e. . The adjusted *p*-values are then given by

Another method was proposed by Benjamini and Yekutieli [11], which is, on the one hand, more conservative, but allows hypotheses to have an arbitrary dependence structure on the other hand. With this BY-method, *p*-values can be adjusted by

A detailed overview of other adjusting methods can be found in Dudoit et al. [12].

### Construction and adjustment of confidence intervals

Using the estimate  for estimating the log fold change of gene *j *as well as the shrunken standard error of this estimate, , the limits of an unadjusted (1 - *α*)-confidence interval can be constructed by

where *t*_1 - *α*/2 _denotes the (1 - *α*/2)-quantile of the *t*-distribution with f* degrees of freedom.

In order to adjust these confidence intervals to coincide with the adjusted *p*-values, the order given by the unadjusted *p*-values *p_j _*needs to be determined, because confidence intervals alone do not reveal information about the strength of significance. Furthermore, adjusted confidence levels need to be determined for constructing the adjusted confidence intervals. Such adjusted confidence levels can be found by considering that the *FDR *can not only be controlled by adjusted *p*-values but also by comparing the unadjusted *p*-values with adjusted significance levels. If we regard again the ordered unadjusted *p*-values,, the adjusted significance level can be found by determining the largest *k *such that . According to the algorithm of Benjamini and Yekutieli [3], we can replace the *α*-level in the above unadjusted confidence intervals by using this *k *in

to obtain BH-adjusted confidence intervals. Similarly, interval limits can be constructed according the BY-method. Here, the adjusted *α*-level is given by

where *k *denotes the largest *k *such that .

### Assessment of biological relevance

Adjusting confidence intervals has direct implications on assessing the potential biological relevance of the genes they belong to. When genes are selected for further laboratory research, not only their *p*-value is compared to a predefined *FDR*-level but also their fold change is compared to some relevance threshold *ρ*, e.g. an absolute log fold change of 1. In this regard we adopt an idea that was first proposed by Jones [9] in the context of clinical trials in COPD and later more generally adopted by Kieser and Hauschke [13], who assessed the clinical relevance of effects by four different categories. In terms of the log fold change for up-regulated genes (and similarly for down-regulated genes), the categories are as follows.

A) *Log fold change statistically significant but not biologically relevant*: The confidence interval lies completely between zero and *ρ*.

B) *Log fold change statistically significant but probably not biologically relevant*: The lower confidence limit is larger than zero, the log fold change lies between zero and *ρ*, and the upper confidence limit exceeds *ρ*.

C) *Log fold change statistically significant and probably biologically relevant*: The lower confidence limit is larger than zero, log fold change and upper confidence limit exceed*ρ*.

D) *Log fold change statistically significant and biologically relevant*: The whole confidence intervals exceeds the threshold *ρ*.

It was also pointed out by Victor [14] that knowledge about the location of a confidence interval relative to a threshold allows a more differentiated interpretation of test results. In the context of selecting genes in a two-group microarray experiment, potentially biologically interesting gene could be missed, if only those genes which are significantly larger than a threshold (i.e., those of category D) are selected. To illustrate this, we will perform a pathway analysis subsequent to categorization. More concrete, we study the association between the genes in the different categories and biological pathways defined by Gene Ontology (GO) terms [15]. A GO term covers information about cellular components, biological processes and molecular functions. Using for example Fisher's exact test, GO-analysis studies whether a certain biological function is associated with more genes among the selected ones than would be expected. We will perform GO-analysis using the R-package topGO [15].

### Simulation settings

In order to analyse the behavior of the *FCR *and the *CCP *when BH- and BY-adjusted confidence intervals are being built within the linear models of Smyth [5], we simulated a typical two group comparison of gene expression data. In particular, the intention of this simulation is to find out whether the findings of Benjamini and Yekutieli [3] on the *FCR *and *CCP *hold under an empirical Bayes approach, i.e. when standard errors are shrinked and confidence intervals are shorter. In each simulation run (totaly 1000), we draw gene expression levels of *m *= 200 genes for 10 samples per group (i.e., *n*_1 _= *n*_2 _= 10) from the multivariate normal distribution *N*(***μ***_*i*_, **∑**). While ***μ***_1 _was always the null vector, ***μ***_1 _= **0***_m_*, *τ *= 25% of randomly selected genes were altered in the second group. In detail, these genes were all altered with the same log fold change *β*. This fold change was varied across the simulations, i.e. *β *was either 0, 1, 2, 3 or 4. The covariance matrix was constructed as follows. In order to mirror the fact that some genes are strongly correlated while other genes are nearly uncorrelated, blocks of 40 genes were assigned a common covariance of either 0, 0.2, 0.4, 0.6 or 0.8:

where ⊗ denotes the Kronecker product and ***J***_*m*/5 _a (*m*/5 × *m*/5)-matrix with all elements being equal 1. In order to include different variances for the genes and to obtain the covariance matrix ∑, the diagonal elements of the (*m *× *m*)-matrix  were than exchanged by a vector of length *m *with elements increasing evenly from 1 to 2. In each simulation run, the *FCR *and the *CCP *were determined.

## Results

### Simulation results

**Figure 1:**
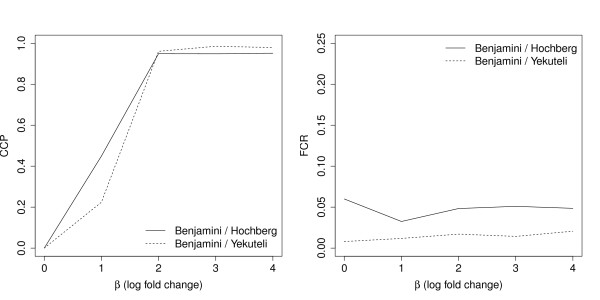
**Simulation results**. Simulated *conditional coverage probability *(*CCP*) and *false coverage-statement rate *(*FCR*) of confidence intervals for the log fold change *β*.

The behavior of the *FCR *and the *CCP *in our simulation study was such as described in a similar setting by Benjamini and Yekutieli [3]. When selecting genes by BH-adjusted *p*-values and constructing the related BH-adjusted confidence intervals, the *CCP *increases with increasing fold changes (Figure 1, left). The same is observed for BY-adjusted confidence intervals. For both methods, the *CCP *is zero, when no genes are altered among the two groups. Thus, the *CCP *is no adequate measure to evaluate the performance of the adjusted confidence intervals.

In contrast, when studying the behavior of the *FCR *(Figure 1, right), it can be observed, that this rate does not seriously depend on the size of the fold change. Furthermore, the rate maintains a level of 5% in essence. Of course, the rate clearly falls below this level when using the more conservative BY-method. Thus, it can also be seen that coverage rate and non-coverage rate are not equivalent when being regarded for genes that are detected as differentially expressed. In summary, the behavior of the *FCR *shows that among all genes detected as differentially expressed, only a small portion of confidence intervals does not cover its true fold change.

### Example data set on rectal cancer

**Figure 2:**
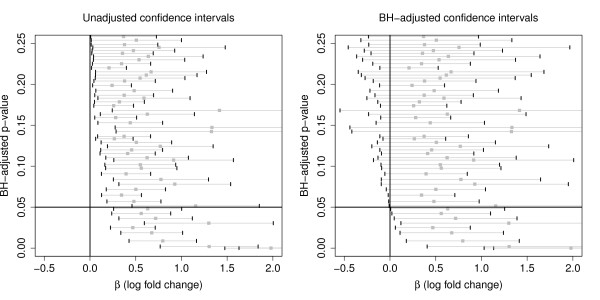
**Unadjusted versus BH-adjusted confidence intervals**. Unadjusted (left) and BH-adjusted (right) confidence intervals, respectively, for up-regulated genes detected in the rectal cancer data, ordered by their corresponding BH-adjusted *p*-values. In order to avoid an overfilling of the plot, only every 50*th *interval is plotted. In the case of adjusted intervals, their lower limit only exceeds zero if the corresponding BH-adjusted *p*-value falls below 0.05.

In order to illustrate the duality of BH-adjusted *p*-values and BH-adjusted confidence intervals we regard microarray data from a rectal cancer study [16]. The data comprises gene expression levels of 33091 genes observed in 79 patients rectal tumors. The data set is publicly available from the ArrayExpress Archive http://www.ebi.ac.uk/arrayexpress/ mainainted by the European Bioinformatics Institute. Patients were clinically classified into *n*_1 _= 12 being lymph node positive and *n*_2 _= 67 being lymph node negative. Using the linear model of Smyth [5] we obtained gene-wise raw *p*-values and adjusted them according the method of Benjamini and Hochberg [1] to control the *FDR*. Among the adjusted *p*-values, 868 were smaller than 5%, indicating the related genes as differentially expressed among the two patient groups. For quantifying the effect of up- or down-regulation, log fold changes were obtained from the linear model fit and their unadjusted 95%-confidence intervals were built. Many of these unadjusted intervals, however, did not coincide with the *p*-value decision. I.e., for many up-regulated genes the lower limit of the related confidence interval exceeded zero, though these genes were not significant according to the FDR-adjusted *p*-values (Figure 2, left). The same can be observed for down-regulated genes in the opposite direction. When, instead, confidence limits are adjusted as proposed by Benjamini and Yekutieli [3], the limits exceed (fall below) zero only if the related BH-adjusted *p*-values indicated significance (Figure 2, right). The formal proof of the duality between adjusted confidence intervals and adjusted *p*-values is given in [3]. By means of this example we illustrated that this principle is also applicable to confidence intervals within an empirical Bayes framework.

### Example data set on lung adenocarcinoma

The misleading character of fold changes without confidence intervals can be observed by another example. Beer et al. [17] studied gene expression levels in samples of 86 patients with lung adenocarcinoma.

**Figure 3:**
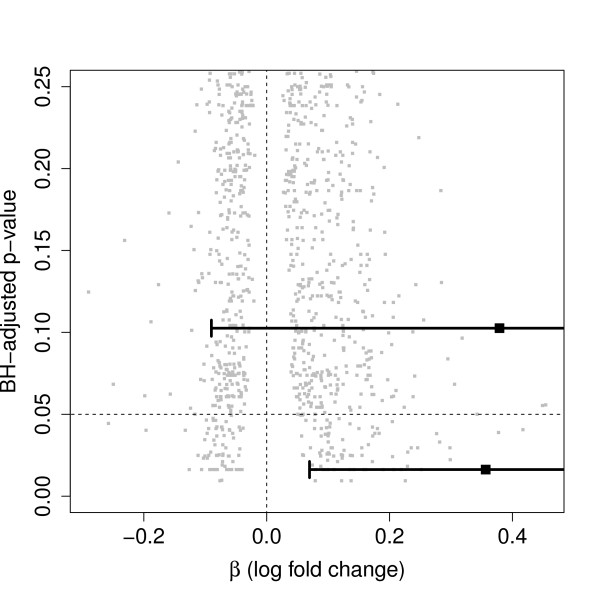
**Significant versus non-significant log fold change**. BH-adjusted *p*-values versus the log fold change *β *derived from the lung cancer data. Two genes with nearly the same log fold change, where the confidence intervals for the one gene expresses significance while the other one does not. Intervals were adjusted according the BH-method.

Sixty-seven (*n*_1_) tumor samples were histopathologically classified as stage I and *n*_2 _= 19 as stage III. The data set contained expression levels 3171 genes measured by oligonucleotide microarrays. We compared these genes among the two tumor stages using the linear models of Smyth [5] and detected 152 genes as differentially expressed according to a false discovery rate of 5%. The gene ranked at place 38 according the adjusted *p*-values has a log fold change of 0.36 and the lower limit for the related BH-adjusted confidence interval exceeds zero. Another gene, ranked at place 411 has actually a slightly larger fold change, 0.38, but it's BH-adjusted confidence interval covers zero (Figure 3). Thus, the adjusted confidence interval can help not to evaluate too optimistically the gene at rank 411. In fact, in this example again, the adjusted confidence intervals coincide with the *p*-value statement.

### Assessment of biological relevance

**Figure 4:**
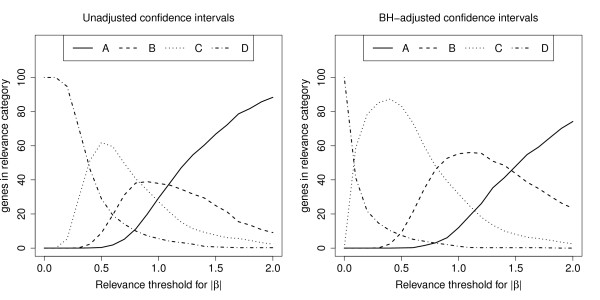
**Biological relevance with regard to unadjusted and adjusted confidence intervals**. Portion (%) of genes in one of four different relevance class versus the threshold for the absolute log fold change (results from the rectal cancer data set).

We studied the distribution of the four categories among all genes in the rectal cancer data example which had an BH-adjusted *p*-values less than 5%, that were 868 genes. Portions of genes in each relevance category are plotted against the relevance threshold *ρ *in Figure 4. The left hand plot displays the case that unadjusted confidence intervals were used. If, in that case, the relevance threshold was chosen to be *ρ *= 0.5, 0% of the significant genes fell into category A, 9% into category B, 62% into category C, and 29% into category D. Thus, 29% of the 868 pre-selected genes (in detail 251) would have been judged to be biologically relevant. The right hand plot displays the case that BH-adjusted confidence intervals were used. Here, only 7% fell into the highest category, yielding 60 genes that would be rated to be biologically relevant.

For 7858 genes of this experiment pathway annotation in terms of Gene Ontologies was available. For each relevance category we tested whether significantly more genes were associated with the different GO terms than would be expected in comparison with the other categories. GO analysis identified 130 GO terms which were significantly associated with genes in category D, 64 with genes in category C, 10 and 23 with genes in categories B and A. I.e., the number of significantly associated GO terms increased with the relevance of the category.

In many microarray experiments the relevance threshold for the log fold change is only loosely established and not fixed before the experiment takes place. Experimenters can employ plots like those shown in Figure 4 to assess how many genes fall in which relevance category given a certain threshold. This might help to find an adequate threshold, which can than be used in related future experiments.

## Discussion

In practice, gene selection in DNA microarray experiments is based on several aspects. It is common that such a group comparison yields several hundreds of significant genes, even with *FDR*-adjusted *p*-values. This result of a multiple testing procedure can, however, only be seen as a first screening. Particularly, gene selection is usually not only based on pure statistical results, i.e. whether a gene is significantly differentially expressed between two distinct groups of samples. Among this bulk of significant genes, those are selected for further laboratory validation which are known to be related to molecular pathways associated with the studied biological system. In addition, the strength of the expression change, in terms of the log fold change, is considered for selecting relevant features. In this context, the potentially divergent statements of *p*-value and log fold change can be confusing for laboratory decision makers. The studied examples in this article have shown, that genes with a large fold change can lack in significance, and vice versa.

One solution to interpret test results and fold changes together are volcano plots, where the log *p*-values are plotted versus log fold changes (see for example Cui and Churchill [18]). Using volcano plots one can easily see which genes are significant on the one hand and highly regulated on the other hand. However, this information gets lost in a tabular representation of test results. Furthermore, a volcano plot cannot be read easily when points overlap. Therefore, we decided to employ confidence intervals which express by their length the high variation behind some genes with a large fold change and can thus help to solve the misleading impression of high relevance.

The four categories of relevance, as proposed by Jones [9] may further assist experimenters to rate the selected genes. In this context, one could eventually replace the term 'biologically relevant' by 'biologically interesting effect size', because larger fold changes might not necessarily have a large impact within a molecular pathway. Performing a GO analysis onto the rectal cancer data data subsequent to differential testing and relevance categorization we could assign pathway information to the different relevance categories. If just volcano plots were employed to assess the biological relevance of significant genes one could perhaps miss interesting pathway information associated with genes of class C ('probably biologically relevant'). With a volcano plot only pathways associated with genes in class D ('biologically relevant') would be taken into account. Thus, the categorization principle opens up insight into gene-pathway associations about those genes which are not highly significant but which may not be completely unimportant as well. Regarding the description of the four categories it should, however, be mentioned that the word 'probably' is only appropriate if the distribution of the log fold change estimates is symmetric. Symmetry is needed to ensure that the estimate exceeds the true value 50% of the time.

**Figure 5:**
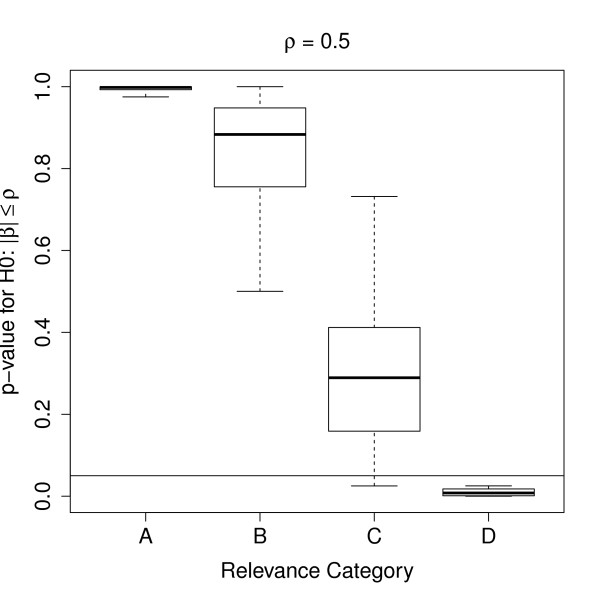
**Testing the log fold change with regard to a threshold versus categorisation into relevance classes**. Comparison of testing the log fold change with regard to a threshold (McCarthy and Smyth [22]) versus categorisation of confidence intervals into relevance classes by our approach. Both methods show a strong correlation. In particular, nearly all significant *p*-values fall in the strongest relevance category D. The comparison was performed using the data of Lips et al. [16] and a relevance threshold of *ρ *= 0.5.

In this article we have combined the ideas of Benjamini and Yekutieli [3] for adjusting confidence intervals with the widely used linear models presented by Smyth [5], in the case of a two group comparison. This new approach of combining these two methods improves a former idea of Jung et al. [4], which was based on unadjusted confidence intervals under normal distribution. Employing the models of Smyth instead, specific characteristics of microarray data are taken into account. In addition, the incorporation of the adjusting algorithm by Benjamini and Hochberg guarantees equal confidence levels for all genes. Using the combination of both methods, confidence intervals can be constructed which coincide with their related *p*-values. I.e., the lower (upper) limits of the confidence intervals for the fold change exceed (falls below) zero, exactly when the *p*-value indicates significance. In a brief simulation we have shown, that the adjusted confidence intervals control a pre-specified *FCR *which was proposed by Benjamini and Yekutieli [3] as reasonable analogue for the *FDR*. More precisely, the simulation results correspond to those in the article by Benjamini and Yekutieli [3]. Thus, we could show that the characteristics of their algorithm are maintained in our combination approach. When we repeated this simulation study with larger numbers of genes (*m *= 1000) and with different portions of differentially expressed features (*τ *= 10% and *τ *= 50%), nearly the same results were obtained. Therefore, we assume that the *FCR *is also independent from the log fold change, even when a larger number of genes is studied like in DNA microarray experiments. An alternative approach to classifying confidence intervals into relevance categories is to test whether log fold changes exceed a given relevance threshold. Methods for this were provided for example by Lewin et al. [19], Bochkina and Richardson [20], van de Wiel and Kim [21] and by McCarthy and Smyth [22]. Using again the data example of Lips et al. [16] and a relevance threshold of *ρ *= 0.5, one can see a strong correlation between the *p*-values derived by the method of McCarthy and Smyth [22] and the classification of confidence intervals into relevance categories by our approach (Figure 5). However, as was seen in the above pathway analysis, certain information about biological pathway information could be missed, when selecting only highly significant genes.

## Conclusions

In summary, our improved combination approach is more adequate for microarray data than a similar approach described previously. Together with the proposed categorization of fold changes it facilitates the selection of genes in microarray experiments and helps to interpret their biological relevance. Although, some mathematical shortcomings of using the *FCR *have been discussed in several published comments (in the same volume as the article of Benjamini and Yekutieli), the practical use of the adjusting algorithm becomes more evident in our examples.

## Competing interests

The authors declare that they have no competing interests.

## Authors' contributions

KJ implemented the method, ran the simulations and worked out the examples. KJ and TB conceived the method and designed the study. TF conceived the idea of the biological relevance criteria. All authors contributed to the writing of the manuscript and read and approved the final manuscript.

## Supplementary Material

Additional file 1 **Example R-code**. Additional file 1 presents an example R-code for calculating adjusted confidence intervals. Click here for file
