# Supplementary material for: Reporting FDR analogous confidence intervals for the log fold change of differentially expressed genes
Source: BMC Bioinformatics. 2011 Jul 15;12:288. doi: 10.1186/1471-2105-12-288 (PMC3154206; doi:10.1186/1471-2105-12-288)
Supplement: Additional file 1 — Example R-code. Additional file 1 presents an example R-code for calculating adjusted confidence intervals. [file 1471-2105-12-288-S1.PDF]

```
library(limma)
library(mvtnorm)

##################################
### Artificial microarray data ###
##################################
d = 500                                     ### Number of genes
n = 10                                      ### Sample per group
mu1 = rep(0, d)                             ### Mean vector group 1
mu2 = rep(0, d)                             ### Mean vector group 2
tau = 0.25                                  ### Portion of differential genes
deg = sample(1:d, tau * d, replace=FALSE)   ### Differential genes
mu2[deg] = 1
Sigma = rbind(                              ### Covariance matrix
c(0.80, 0.60, 0.40, 0.20, 0.00),
c(0.60, 0.80, 0.60, 0.40, 0.20),
c(0.40, 0.60, 0.80, 0.60, 0.40),
c(0.20, 0.40, 0.60, 0.80, 0.60),
c(0.00, 0.20, 0.40, 0.60, 0.80))
Sigma = Sigma %x% matrix(1, d/5, d/5)
diag(Sigma) = seq(1, 2, length.out=d)       ### Variances of genes
X1 = t(rmvnorm(n, mu1, Sigma))              ### Expression levels group 1
X2 = t(rmvnorm(n, mu2, Sigma))              ### Expression levels group 2
X = cbind(X1, X2)

#############
### Limma ###
#############
group = gl(2, n)
design = model.matrix(~ group)
fit1 = lmFit(X, design)
fit = eBayes(fit1)
p.un = fit$p.value[,2]                      ### Unadjusted p-values
p.bh = p.adjust(p.un, method="BH")          ### BH-adjusted p-values
p.by = p.adjust(p.un, method="BY")          ### BY-adjusted p-values

#######################################
### Unadjusted confidence intervals ###
#######################################
alpha = 0.05                                ### Significance level
beta = fit$coefficients[,2]
std = sqrt(fit$s2.post) * sqrt(fit$cov.coefficients[2,2])
dof = fit$df.prior + fit$df.residual[1]
cl = 1 - alpha / 2
lower.un = beta - qt(cl, dof) * std         ### Lower confidence limits
upper.un = beta + qt(cl, dof) * std         ### Lower confidence limits

########################################
### BH-adjusted confidence intervals ###
########################################
R.deg = length(which(p.bh < alpha))
cl = 1 - (R.deg * alpha / d) / 2
lower.bh = beta - qt(cl, dof) * std
upper.bh = beta + qt(cl, dof) * std

########################################
### BY-adjusted confidence intervals ###
########################################
R.deg = length(which(p.by < alpha))
cl = 1 - (R.deg * alpha / d) / 2
lower.by = beta - qt(cl, dof) * std
upper.by = beta + qt(cl, dof) * std
```
